# Quality assessment of kidney cancer clinical practice guidelines using AGREE II instrument: A critical review

**DOI:** 10.1097/MD.0000000000017132

**Published:** 2019-10-04

**Authors:** XiaoFeng Hou, Meixuan Li, Wenbo He, Meng Wang, Peijing Yan, Caiwen Han, Huijuan Li, Liujiao Cao, Biao Zhou, Zhenxing Lu, Bibo Jia, Jing Li, Xu Hui, Yunxia Li

**Affiliations:** a Gansu Provincial Cancer Hospital; b School of Public Health, Lanzhou University; c Evidence Based Medicine Center, School of Basic Medical Sciences, Lanzhou University; d Institute of Clinical Research and Evidence Based Medicine, The Gansu Provincial Hospital; e Department of Clinical Medicine, Gansu University of Traditional Chinese Medicine; f The First Clinical Medical College, Lanzhou University; g Department of Oncology, Gansu Gem Flower Hospital, Lanzhou, China.

**Keywords:** AGREE II, clinical practice guidelines, kidney cancer, quality assessment

## Abstract

**Background:**

Evidence-based guidelines are expected to provide clinicians with explicit recommendations on how to manage health conditions and bridge the gap between research and clinical practice. However, the existing practice guidelines(CPGs) vary in quality. This study aimed to evaluate the quality of CPGs of kidney cancer.

**Methods:**

We systematically searched PubMed, Embase, China Biology Medicine disc, and relevant guideline websites from their inception to April, 2018. We identified CGPs that provided recommendations on kidney cancer; 4 independent reviewers assessed the eligible CGPs using the Appraisal of Guidelines for Research and Evaluation (AGREE II) instrument. The consistency of evaluations was calculated using intraclass correlation coefficients (ICC).

**Results:**

A total of 13 kidney cancer CGPs were included. The mean scores for each AGREEII domain were as follows: scope and purpose—76.9%; clarity and presentation—76.4%; stakeholder involvement—62.8%; rigor of development—58.7%; editorial independence—53.7%; and applicability—49.4%. Two CPGs were rated as “recommended”; 8 as “recommended with modifications”; and 3 as “not recommended.” Seven grading systems were used by kidney cancer CGPs to rate the level of evidence and the strength of recommendation.

**Conclusions:**

Overall, the quality of CPGs of kidney cancer is suboptimal. AGREE II assessment results highlight the need to improve CPG development processes, editorial independence, and applicability in this field. It is necessary to develop a standardized grading system to provide clear information about the level of evidence and the strength of recommendation for future kidney cancer CGPs.

## 1 Introduction

An estimated 62,700 Americans were diagnosed with kidney cancer and 14,240 died of the disease in 2016.^[1]^ The vast majority (greater than 90%) of kidney cancers are renal cortical tumors known as renal cell carcinoma (RCC).^[2]^ RCC comprises approximately 3.8% of all new cancers in the western world; the detection rate of RCC has been increasing in the past 10 years by approximately 1.7% per year.^[3]^ Since 2005, a number of new targeted agents have come into the market for the treatment of this disease.^[4]^ Although many of these therapies showing promising outcomes with improved progression-free survival and overall survival, diagnosis, treatment, and management of kidney cancer still remain the major challenge for clinicians.

Therefore, kidney cancer clinical practice guidelines (CPGs) drafted by local, national, and international organizations have been developed to standardize clinical practice and improve effectiveness of management. Ideally, evidence-based guidelines are expected to provide clinicians with explicit recommendations on how to manage health conditions and bridge the gap between research and clinical practice.^[5]^ However, the existing CPGs vary in quality and comprehensiveness, leading to difficulties with standardization of care, adaptation, and implementation, particularly in resource-limited settings. The usefulness of guidelines primarily depends on the quality, rigorous methodology, and transparency of development.^[6]^ It is important to determine whether the recommendations are, indeed, based on high-quality evidence.^[7,8]^ At present, there is no literature comparing and evaluating the strengths and weaknesses of all available CPGs for the treatment of kidney cancer.

We aimed to assess and summarize the quality of all currently available international kidney cancer CPGs by conducting a critical review using the Appraisal of Guidelines for Research and Evaluation (AGREE) II instrument.^[9]^ We sought to identify gaps limiting evidence-based practice, and highlight potential opportunities for improvement.

## 2 Materials and methods

We conducted a comprehensive evaluation of kidney cancer CPGs using the AGREE II instrument, and the study was performed according to the guidelines from Preferred Reporting Items for Systematic Reviews and Meta-analyses (PRISMA)^[10]^ and some related studies.^[11–13]^ As it is a review of the previous works of literature, approval of the ethics committee was not required.

### 2.1 Search strategy

PubMed, Embase, and China Biology Medicine disc databases were systematically searched up to April, 2018. We combined the terms “kidney cancer,” “renal cell carcinoma,” “renal tumor,” and a filter to identify guideline documents (practice guideline [pt] OR guideline [pt] OR guideline∗ [ti]). We also searched the websites of guideline development organizations: Guidelines International Network Web site (http://www.g-i-n.net/), National Institute for Health for Health and Care Excellence website (https://www.nice.org.uk/guidance), National Guideline Clearinghouse (https://guidelines.gov/), Scottish Intercollegiate Guidelines Network (http://www.sign.ac.uk/), Clinical Practice Guidelines Portal website (https://www.clinicalguidelines.gov.au/), New Zealand Guidelines Group website (https://www.health.govt.nz/), BCGuidelines website (http://www.bcguidelines.ca/alphabetical), AQuMed Database website (http://www.aezq.de/aezq/publications). In addition, we searched Google Search Engine and checked the references of all the related guidelines to include more potential guidelines.

### 2.2 Inclusion and exclusion criteria

The inclusion criteria were as follows: complete guideline text is available in English; guideline contains recommendations regarding kidney cancer interventions; and the guideline should be published after 2008. If the guideline had been updated, only the most recent version was assessed. For every guideline ultimately included, we thoroughly searched for accompanying technical and supporting documents to better inform our assessments. The following studies will be excluded: duplicate guidelines, guidelines for patients, editorials, secondary or multiple publications, and short summaries.

### 2.3 Guideline screening and data extraction

Two authors (L.M.X. and Y.P.J.) independently identified search results to determine eligibility guidelines, and extracted the basic information from included guidelines. Disagreements were resolved by consulting the third expert adjudicator (L.Y.X.).

### 2.4 Quality appraisal of guidelines

Four independent reviewers evaluated the quality of each kidney cancer CPG according to AGREE II instrument,^[14]^ which includes 23 items on a 7-point Likert scale across 6 domains. Each domain captures a unique dimension of the CPG quality: scope and purpose, stakeholder involvement, rigor of development, clarity and presentation, applicability, and editorial independence. Items were scored based on a scale ranging from 1 (absence of item) to 7 (item is reported with exceptional quality). The standardized score for individual domain, which ranged from 0% to 100%, was calculated using the following formula: (actual score − minimal possible score)/(maximal possible score − minimal possible score) × 100%. AGREE II protocol^[14]^ states that no overall score is calculated to determine if a CPG is recommended or not recommended. Each guideline was classified as: “recommended” for overall scores >60%, “recommended with modifications” for scores between 30% and 60%, and “not recommended” for scores <30%.^[15]^

### 2.5 Strength of recommendation and level of evidence

We extracted the level of evidence and the strength of recommendations of each kidney cancer guideline if they adopted evidence grading systems.

### 2.6 Statistical analysis

We calculated the standardized score of each domain for individual included CPGs, and determined the number of recommendations and the percentage distributions among quality of evidence and strength of recommendation classes. Agreement among 4 appraisers’ scores was tested using intraclass correlation coefficients (ICCs) with 95% confidence interval (CI) for each domain of all included guidelines.^[16]^ As a previous study described,^[17]^ the ICCs between 0.01 and 0.20 were considered minor, 0.21 to 0.40 fair, 0.41 to 0.60 moderate, 0.61 to 0.80 substantial, and 0.81 to 1.00 very good. A value of *P* <.05 indicated statistical significance. All tests were 2-sided. Statistical analyses were conducted using Excel2010 and SPSS version 21.0 (SPSS Inc., Chicago, IL).

## 3 Results

### 3.1 Study selection

**Figure 1:**
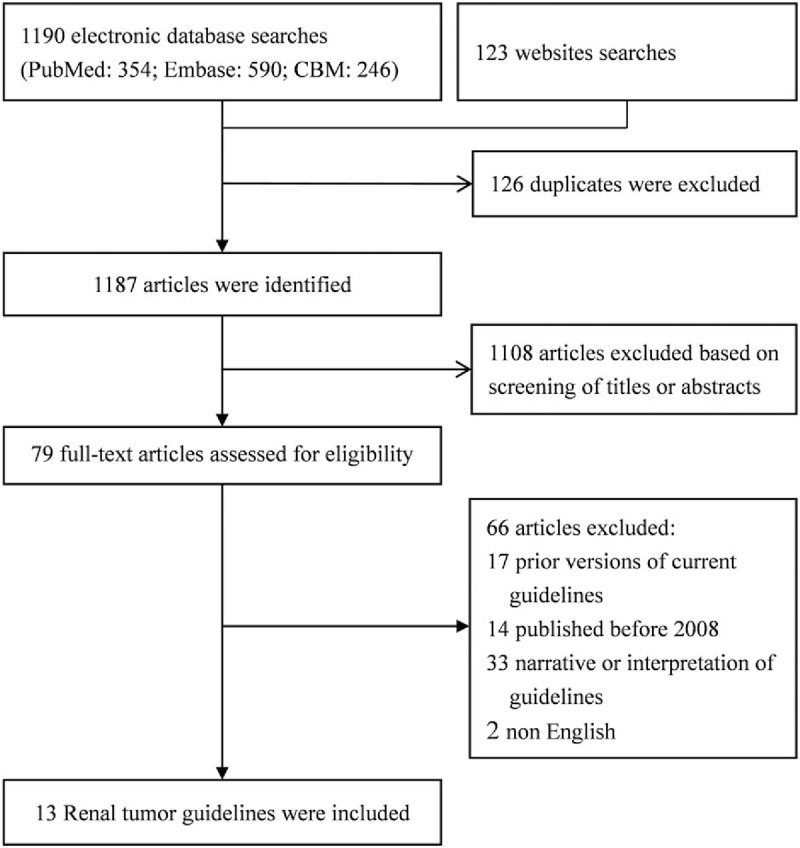
Flowchart of kidney cancer guidelines searching and selection.

Figure 1 shows the flow how we identified and selected the guidelines. The initial search yielded 1313 titles and abstracts, of which 126 were excluded as duplicates and 1108 were removed after reviewing abstracts. Full text identified was then performed on a total of 79 articles, of which only 13^[2,4,18–28]^ met inclusion criteria.

### 3.2 CPG characteristics

A summary of the characteristics of the included CPGs was presented in Table 1. Thirteen kidney cancer guidelines were included in our study representing 12 different organizations and spanning several countries. Of these 13 CPGs, 6^[2,4,18,20,21,28]^ were new, and the rest were updates; 12^[2,4,18–28]^ were developed in high-income countries and only 1^[23]^ was from middle-income country(China). The CPGs evaluated covered the different types of kidney cancers: 8 guidelines^[4,20,22–25,27,28]^ focus on RCC, 3^[2,21,26]^ for renal mass and localized renal cancer, and 2^[18,19]^ for all stages of kidney cancer. The majority (8) of CPGs focused on the early management of kidney cancer,^[2,18,19,21–23,25,28]^ and others^[4,20,24,26,27]^ focused on the diagnosis, treatment, and follow-up.

**Table 1:**
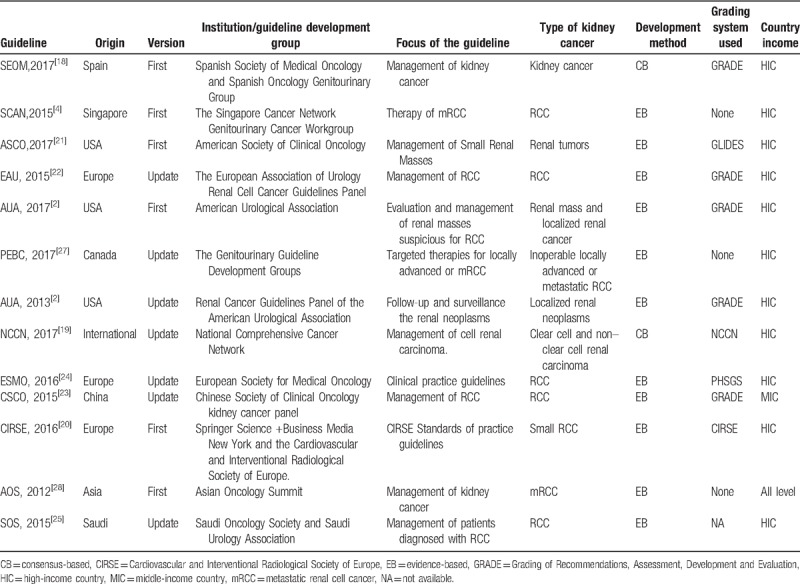
The characteristics of included kidney cancer guidelines.

### 3.3 CPG quality assessment (AGREE)

#### 3.3.1 Consistency

The ICC values indicated that the overall agreement among 4 appraisers received higher reliability scores, ranging from 0.57 to 0.92 (Table 2). The ICCs for the AGREE appraisal conducted by the 4 reviewers was lowest in the “applicability” domain (0.57), highest in the “rigor of development” domain (0.92), and the overall assessment was 0.79, which indicated the intrareviewer item score agreement was good. Domain scores of the AGREE II quality assessment are illustrated in Table 2.

**Table 2:**
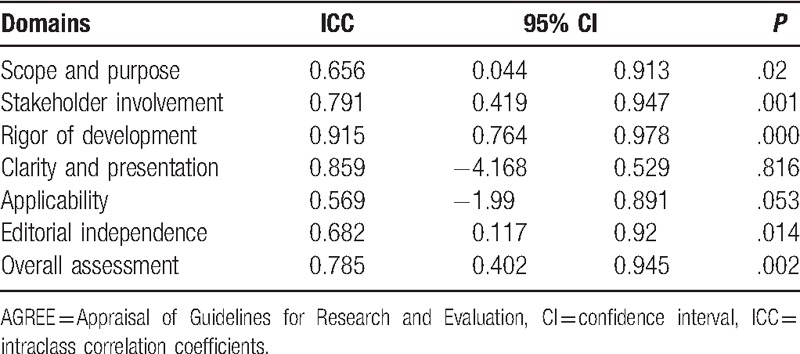
Inter-rater reliability for each AGREE quality domain.

#### 3.3.2 Domain 1: scope and purpose

This domain includes the main objectives of the CPGs, the health questions, and the target population. The mean score of kidney cancer GPGs in this domain is 76.9%, with a standard deviation (SD) of 9.5%, and all guidelines scored more than 50%. The lowest score was 63%, from SEOM clinical guideline for treatment of kidney cancer 2017 (SEOM, 2017). The highest score was 90.3%, from the use of targeted therapies in patients with inoperable locally advanced or metastatic renal cell cancer: updated guideline 2017 (PEBC, 2017).

#### 3.3.3 Domain 2: stakeholder involvement

This domain focuses on the extent to which the CPG was developed by the appropriate stakeholders and represents the views of its intended users. Scores fluctuated remarkably with a mean score ± SD of 62.85% ± 17.4%. Two (15.4%) kidney cancer guidelines scored lower than 50%, of which the lowest was 24% from SEOM (SEOM, 2017).

#### 3.3.4 Domain 3: rigor of development

This domain investigates the method and process of evidence search, grading, summary, and the formulation of the recommendations. The mean score and SD for this domain was 58.7% ± 18.4%. Three (23.1%) kidney cancer guidelines scored lower than 50%, of which the lowest was 27% from Saudi Oncology Society and Saudi Urology Association combined clinical management guidelines (SOS, 2015).

#### 3.3.5 Domain 4: clarity of presentation

This domain addresses the presentation and format of guidelines. The mean score and SD in this domain was 76.4% ± 13.8%. The lowest score was 50% from Saudi Oncology Society and Saudi Urology Association combined clinical management guidelines for renal cell carcinoma (SOS, 2015).

#### 3.3.6 Domain 5: Application

This domain focuses on processes related to CPG implementation such as organizational facilitators and barriers, additional materials provided, cost implications, and monitoring or audit criteria. The mean score and SD of this domain was 49.4% ± 21.6%, among which 4 kidney cancer guidelines scored less than 50%, with the lowest score of 3% from SEOM clinical guideline for treatment of kidney cancer (2017) (SEOM, 2017).

#### 3.3.7 Domain 6: editorial independence

This domain considers funders and competing interests of experts involved in guideline development. The mean score and SD of this domain was 53.7% ± 18.1%, and 5 scored less than 50%. The lowest score of 25% came from Cardiovascular and Interventional Radiological Society of Europe (CIRSE) guidelines on percutaneous ablation of small renal cell carcinoma (CIRSE, 2016) and SEOM clinical guideline for treatment of kidney cancer (SEOM, 2017). The highest score was 79.2%, from European Association of Urology Guidelines on Renal Cell Carcinoma 2015 (EAU, 2015).

#### 3.3.8 Overall assessment

This assessment concerns “the rating of body quality of the guidelines and whether the guideline would be recommended for use in practice.” According to the appraisal of the individual domains and overall scores, 2 kidney cancer guidelines overall scored >60%, and were rated as “recommended” by the appraisers; 8 were rated as “recommended with modifications”; and 3 as “not recommended” (Table 3).

**Table 3:**
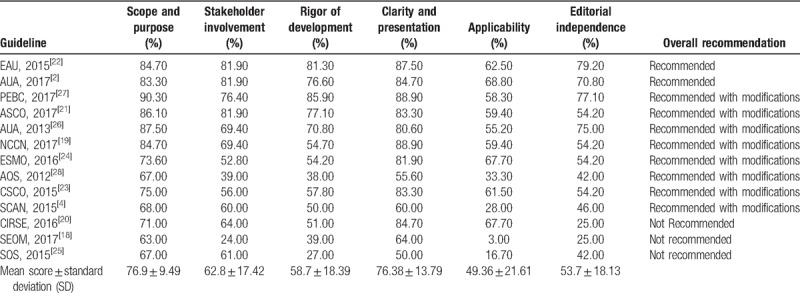
AGREE score by domain of each kidney cancer guideline.

#### 3.3.9 Level of evidence and strength of recommendation

Of the 13 included kidney cancer guidelines, 11^[2,4,20–28]^ of them were deemed evidence-based and 2^[18,19]^ were deemed expert consensus-based. Ten guidelines used grading systems to rate the level of evidence and the strength of recommendation, among which 3^[2,23,26]^ adopted Grading of Recommendations, Assessment, Development and Evaluation (GRADE) system (AUA,2017; AUA,2013; CSCO,2015), 1 ^[21]^ used GLIDES system (ASCO, 2017), 1^[19]^ used NCCN system (NCCN, 2017), 1^[24]^ used PHSGS system (ESMO, 2016), 1^[20]^ used CIRSE system (CIRSE, 2016), and 3^[18,22,25]^ did not specify(SEOM, 2017; EAU, 2015; SOS, 2015). Whereas, the codes of level of evidence and strength of recommendation in different grading systems vary (Table 4  ).

**Table 4:**
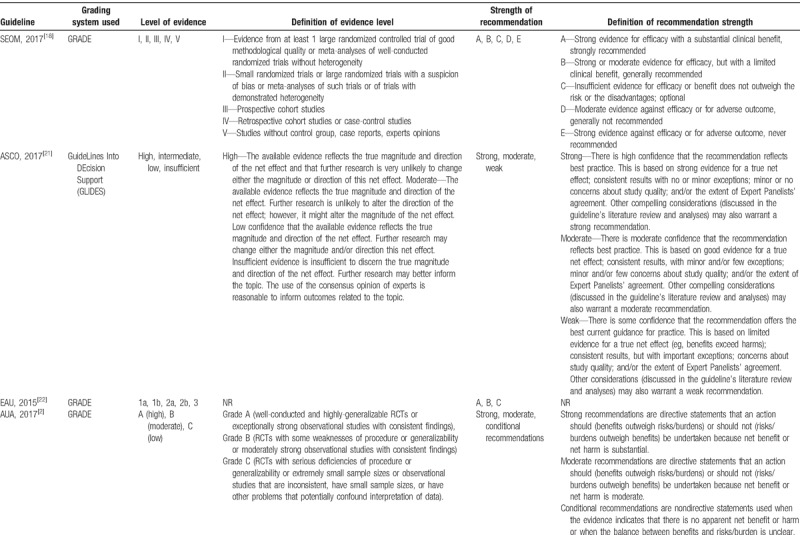
Grading systems used in the included guidelines.

**Table 4 (Continued):**
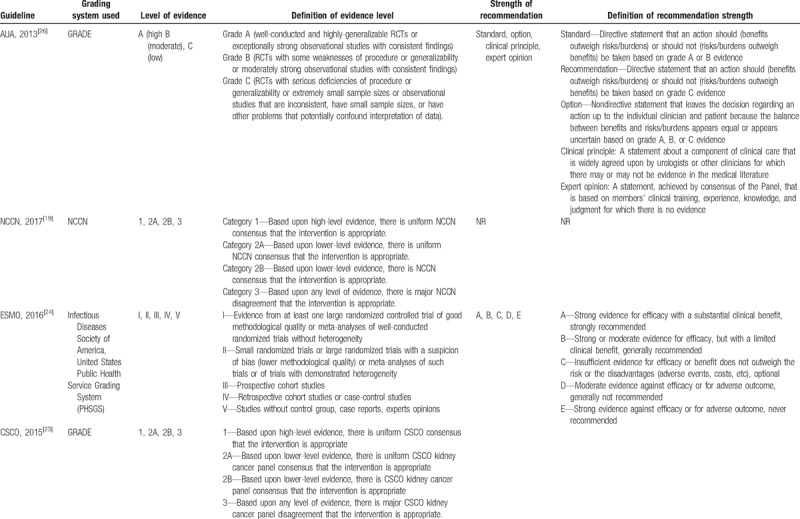
Grading systems used in the included guidelines.

**Table 4 (Continued):**
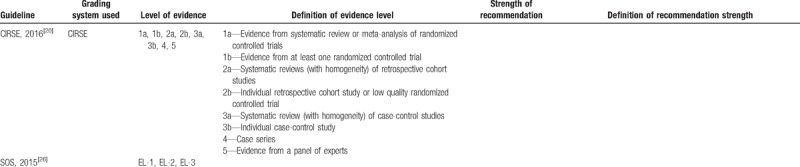
Grading systems used in the included guidelines.

## 4 Discussion

The study evaluated the quality of kidney cancer CPGs published after 2008, and 13 kidney cancer CPGs were included. Two guidelines were rated as “recommended,” 8 as “recommended with modifications,” and 3 as “not recommended.” Seven grading systems were used by kidney cancer CGPs to rate the level of evidence and the strength of recommendation.

There may exist some kidney cancer CPGs published before 2008,^[29,30]^ and were not updated, but the recommendations in those guidelines had been outdated and could not be used in practice according to IOM statements of CPGs.^[31]^ Hence, we did not include these CPGs in this review. Among the 13 kidney cancer CPGs included, the highest mean scores were achieved in scope and purpose, stakeholder involvement, and clarity and presentation, whereas the main weaknesses across kidney cancer CPGs were rigor of development, applicability, and editorial independence. The European Association of Urology Guidelines on Renal Cell Carcinoma 2015 (EAU, 2015), The Use of Targeted Therapies in Patients with Inoperable Locally Advanced or Metastatic Renal Cell Cancer: Updated Guideline 2017 (PEBC, 2017), and Renal mass and localized renal cancer: AUA guideline (AUA, 2017) were the 3 CPGs with best results. Most of the included CPGs were developed by high-income countries, and are therefore minimally applicable in resource-limited settings. Apart from this, the distribution of level of evidence and strength of recommendations varied significantly among different kidney cancer CPGs.

The appraisal CPGs obtained the lowest score in *applicability* domain, suggesting that guideline developers have not paid sufficient attention to potential barriers affecting practical implementation of recommendations. Therefore, it is recommended that there should be a pilot test for the applicability of new guidelines before the release of clinical practice to ensure their feasibility. Guideline groups should provide recommendations and address the barriers as much specificity as the evidence permits.^[32]^ The guideline developed by AUA (2017) ^[2]^ was recommended (scoring 68.8%) in our appraisal as a good example in future guideline development for this domain.

Kidney cancer CPGs also performed poorly in *editorial independence* domain, information related to potential conflicts of interest was scarce or not even mentioned, especially the guidelines developed by SEOM, CIRSE, SOS, and AOS. Because the conflicts of interest are the most common source of bias and often under-reported, CPG developers should explicitly declare whether potential conflicts of interest (such as between editorial board and pharmaceutical or medical device manufacturer) will impact on guideline drafting, including the rigorous vetting process and the transparent and available rules for review. Recently, some studies have reported that developers of CPGs were affected by pharmaceutical or medical device manufacturers, so it is important to know how much these interactions could have affected the recommendations.^[33,34]^

Rigor of CPGs mainly focuses on the methodological process of guidelines development, because this domain can better reflect the quality of CPGs than the other 5 domains. Even though vast majority of guidelines contained references, many did not explicitly describe literature search and selection methods, and were ambiguous regarding how to appraise evidence and formulate recommendations. This step is crucial to determine whether the recommendations really depend on the best available evidence. The low score might be caused by the poor methodology and reporting, or unfamiliarity with criteria of CPG development, or missing performance of external peer review and updating process.

As we all know, adaptation of existing guidelines to clinical practice may be a more valid and cost-effective means of achieving high-quality guidelines worldwide.^[35]^ To achieve this aim, the majority of guidelines applied grading systems to rate the quality of evidence so as to communicate clear message, quickly and concisely to help guideline users, readers, and stakeholders to understand the confidence of estimate of the effects and the strength of recommendations. The confidence of estimate of the effects reflects the extent to which confidence in an estimate of the effect is adequate to support a particular recommendation. Also, the strength of guideline recommendation reflects the extent of collective confidence that adherence to the recommendation will do more good than harm.^[36,37]^ However, we found different grading systems with various systems of codes were used to rate evidence and recommendations in kidney cancer CPGs, which could confuse the guideline users to apply these guidelines. Therefore, it is important to develop a standardized grading system to provide clear information about the level of evidence and the strength of recommendation for kidney cancer CPG users, and good news is that we find some guideline organizations such as the American Urological Association (AUA) begin to adopt GRADE system instead of old systems in their new version of guideline development handbooks.^[2,26]^

There are several strengths of our findings. On the one hand, the strength of recommendations and level of evidence of each kidney cancer guideline were carefully extracted if these guidelines adopted evidence grading systems, which may indicate the overall quality of kidney cancer guidelines; On the other hand, our authors have different academic backgrounds, including methodological and medical experts, which ensured the reliability of our conclusions.

Inevitably, our study has some limitations: Firstly, we only included guidelines published in English; guidelines for some other languages are not included and may affect the universality of the results. Secondly, AGREE II instrument places emphasis on methods of guideline development and the transparency of reporting, but could not assess potential impacts of recommendations on patient outcomes.^[38,39]^

## 5 Conclusions

Our analysis of current CPGs for the kidney cancer revealed that methodological quality of CPGs was acceptable, but there is still plenty of space for improvement, especially in the editorial independence, applicability, and rigor of development in the CPG development. Kidney cancer CPGs should develop recommendations with the evidence of high quality, while minimizing bias with compelling methodological rigor, openness, and transparency. If possible, CPGs should underline the demand for additional studies to close the gaps in clinical care that has a significant effect on patient outcomes.

## Author contributions

**Data curation:** Xiaofeng Hou, Meixuan Li, Peijing Yan, Meng Wang, Wenbo He, Caiwen Han, Huijuan Li, Liujiao Cao, Biao Zhou, Zhenxing Lu, Bibo Jia, Jing Li, Xu Hui.

**Formal analysis:** Xiaofeng Hou, Meixuan Li, Peijing Yan, Meng Wang, Wenbo He.

**Methodology:** Xiaofeng Hou, Meixuan Li, Peijing Yan, Yunxia Li.

**Project administration:** Yunxia Li.

**Resources:** Caiwen Han, Huijuan Li, Liujiao Cao, Biao Zhou, Zhenxing Lu, Bibo Jia, Jing Li, Xu Hui.

**Software:** Wenbo He, Huijuan Li.

**Supervision:** Yunxia Li.

**Writing – original draft:** Xiaofeng Hou, Meixuan Li, Meng Wang.

**Writing – review & editing:** Xiaofeng Hou, Meixuan Li, Peijing Yan, Yunxia Li.

## References

[1] Siegel RL Miller KD Jemal A Cancer statistics, 2016. CA Cancer J Clin 2016;66:7–30. 26742998 10.3322/caac.21332

[2] Campbell S Uzzo RG Allaf ME Renal mass and localized renal cancer: AUA guideline. J Urol 2017;198:520–9. 28479239 10.1016/j.juro.2017.04.100

[3] http://seer.cancer.gov/statfacts/html/kidrp.html Accessed November, 2015.

[4] Kanesvaran R Ng QS Tan MH Singapore cancer network (SCAN) guidelines for systemic therapy of metastatic renal cell carcinoma (mRCC). Ann Acad Med Singapore 2015;44:406–14. 26763058

[5] Bero LA Grilli R Grimshaw JM Closing the gap between research and practice: an overview of systematic reviews of interventions to promote the implementation of research findings. The Cochrane Effective Practice and Organization of Care Review Group. BMJ 1998;317:465–8. 9703533 10.1136/bmj.317.7156.465 PMC1113716

[6] Grol R Cluzeau FA Burgers JS Clinical practice guidelines: towards better quality guidelines and increased international collaboration. Br J Cancer 2003;89 suppl 1:S4–8. 12915896 10.1038/sj.bjc.6601077 PMC2753001

[7] Isaac A Saginur M Hartling L Quality of reporting and evidence in American Academy of Pediatrics guidelines. Pediatrics 2013;131:732–8. 23530180 10.1542/peds.2012-2027

[8] Khan AR Khan S Zimmerman V Quality and strength of evidence of the Infectious Diseases Society of America clinical practice guidelines. Clin Infect Dis 2010;51:1147–56. 20946067 10.1086/656735

[9] Brouwers M Kho M Browman G AGREE II: advancing guideline development, reporting and evaluation in health care. CMAJ 2010;182:E839–42. 20603348 10.1503/cmaj.090449 PMC3001530

[10] Moher D Liberati A Tetzlaff J the PRISMA Group. Preferred Reporting Items for Systematic Reviews and Meta-analyses: the PRISMA statement. PLoS Med 2009;6:e1000097. 19621072 10.1371/journal.pmed.1000097 PMC2707599

[11] Di BS Wei M Ma WJ A critical review to traumatic brain injury clinical practice guidelines. Medicine (Baltimore) 2019;98:e14592. 30817576 10.1097/MD.0000000000014592 PMC6831439

[12] Li L Tian JH Tian HL Network meta-analyses could be improved by searching more sources and by involving a librarian. J Clin Epidemiol 2014;67:1001–7. 24841794 10.1016/j.jclinepi.2014.04.003

[13] Yao L Sun R Chen YL The quality of evidence in Chinese meta-analyses needs to be improved. J Clin Epidemiol 2016;74:73–9. 26780259 10.1016/j.jclinepi.2016.01.003

[14] Brouwers MC Kho ME Browman GP AGREE Next Steps Consortium. AGREE II: advancing guideline development, reporting and evaluation in health care. CMAJ 2010;182:E839–842. 20603348 10.1503/cmaj.090449 PMC3001530

[15] Jiang M Guan WJ Fang ZF A critical review of the quality of cough clinical practice guidelines. Chest 2016;150:777–88. 27164291 10.1016/j.chest.2016.04.028

[16] Shrout P Fleiss J Intraclass correlations: uses in assessing rater reliability. Psychol Bull 1979;86:420–8. 18839484 10.1037//0033-2909.86.2.420

[17] Alonso-Coello P Irfan A Sola I The quality of clinical practice guidelines over the last two decades: a systematic review of guideline appraisal studies. Qual Saf Health Care 2010;19:e58. 21127089 10.1136/qshc.2010.042077

[18] Gallardo E Mendez-Vidal MJ Perez-Gracia JL SEOM clinical guideline for treatment of kidney cancer (2017). Clin Transl Oncol 2018;20:47–56. 29134564 10.1007/s12094-017-1765-4 PMC5785618

[19] Motzer RJ Jonasch E Agarwal N kidney cancer, version 2.2017, NCCN Clinical Practice Guidelines in Oncology. J Natl Compr Canc Netw 2017;15:804–34. 28596261 10.6004/jnccn.2017.0100

[20] Krokidis ME Orsi F Katsanos K CIRSE guidelines on percutaneous ablation of small renal cell carcinoma. Cardiovasc Intervent Radiol 2017;40:177–91. 27987000 10.1007/s00270-016-1531-y

[21] Finelli A Ismaila N Bro B Management of small renal masses: American Society of Clinical Oncology clinical practice guideline. J Clin Oncol 2017;35:668–80. 28095147 10.1200/JCO.2016.69.9645

[22] Powles T Staehler M Ljungberg B European Association of Urology guidelines for clear cell renal cancers that are resistant to vascular endothelial growth factor receptor-targeted therapy. Eur Urol 2016;70:705–6. 27349614 10.1016/j.eururo.2016.06.009

[23] Guo J Ma J Sun Y Chinese guidelines on the management of renal cell carcinoma (2015 edition). Chin Clin Oncol 2016;5:12. 26932436 10.3978/j.issn.2304-3865.2015.11.01

[24] Escudier B Porta C Schmidinger M Renal cell carcinoma: ESMO clinical practice guidelines for diagnosis, treatment and follow-up. Ann Oncol 2016;27:v58–68. 27664262 10.1093/annonc/mdw328

[25] Alghamdi A Alkhateeb S Alghamdi K Saudi Oncology Society and Saudi Urology Association combined clinical management guidelines for renal cell carcinoma. Urol Ann 2016;8:136–40. 27141180 10.4103/0974-7796.179239 PMC4839227

[26] Donat SM Diaz M Bishoff JT Follow-up for clinically localized renal neoplasms: AUA guideline. J Urol 2013;190:407–16. 23665399 10.1016/j.juro.2013.04.121

[27] The Use of Targeted Therapies in Patients with Inoperable Locally Advanced or Metastatic Renal Cell Cancer: Updated Guideline 2017. Available at: https://www.guideline.gov/summaries/summary/51032/the-use-of-targeted-therapies-in-patients-with-inoperable-locally-advanced-or-metastatic-renal-cell-cancer-updated-guideline-2017?q=The+Use+of+Targeted+Therapies+in+Patients+with+Inoperable+Locally+Advanced+or+Metastatic+Renal+Cell+Cancer.

[28] Chiong E Tay MH Tan MH Management of kidney cancer in Asia: resource-stratified guidelines from the Asian Oncology Summit 2012. Lancet Oncol 2012;13:e482–91. 23117003 10.1016/S1470-2045(12)70433-3

[29] Nakamoto T Treatment guideline of renal cancer. Jpn J Clin Urol 2003 559–63.

[30] Miller K Renal cell carcinoma. Guidelines for diagnosis and treatment. Urol Int 1999;63:6–9. 10592483 10.1159/000030411

[31] National Academies Press; 2011;Steinberg E Greenfield S Wolman DM Mancher M Graham R Clinical Practice Guidelines We Can Trust. 24983061

[32] Brouwers MC Kho ME Browman GP AGREE Next Steps Consortium. AGREE II: advancing guideline development, reporting, and evaluation in healthcare. CMAJ 2010;182:E839–842. 20603348 10.1503/cmaj.090449 PMC3001530

[33] Lexchin J Bero LA Djulbegovic B Pharmaceutical industry sponsorship and research outcome and quality: systematic review. BMJ 2003;326:1167–70. 12775614 10.1136/bmj.326.7400.1167 PMC156458

[34] Hurwitz B Clinical guidelines: legal and political considerations of clinical practice guidelines. BMJ 1999;318:661. 10066215 10.1136/bmj.318.7184.661 PMC1115098

[35] Fervers B Burgers J Haugh M Adaptation of clinical guidelines: literature review and proposition for a framework and procedure. Int J Qual Health Care 2006;18:167–76. 16766601 10.1093/intqhc/mzi108

[36] Atkins D Best D Briss PA Grading quality of evidence and strength of recommendations. BMJ 2004;328:1490. 15205295 10.1136/bmj.328.7454.1490 PMC428525

[37] Guyatt GH Oxman AD Vist GE GRADE Working Group. GRADE: an emerging consensus on rating quality of evidence and strength of recommendations. BMJ 2008;336:924–6. 18436948 10.1136/bmj.39489.470347.AD PMC2335261

[38] Vlayen J Aertgeerts B Hannes K A systematic review of appraisal tools for clinical practice guidelines: multiple similarities and one common deficit. Int J Qual Health Care 2005;17:235–42. 15743883 10.1093/intqhc/mzi027

[39] Watine J Friedberg B Nagy E Conflict between guideline methodologic quality and recommendation validity: a potential problem for practitioners. Clin Chem 2006;52:65–72. 16391328 10.1373/clinchem.2005.056952

